# Angio-Behçet thoracique - régression totale sous traitement immuno-suppresseur (à propos d'un cas)

**DOI:** 10.11604/pamj.2014.18.116.3383

**Published:** 2014-06-05

**Authors:** Rhizlane Berrady, Zineb Khammar, Mariam Lahlou, Amal Boukhrissa, Wafaa Bono

**Affiliations:** 1 Service de médecine interne CHU Hassan II, Fes, Maroc

**Keywords:** Maladie de Behçet, traitement immuno-suppresseur, anévrisme, Behcet's disease, immuno-suppressive therapy, aneurism

## Abstract

La maladie de Behçet est une Vascularite multisystémique d’étiologie obscure. L'angio-Behçet thoracique est en rapport avec le tropisme vasculaire bien connu de la maladie, et les anévrysmes des artères pulmonaires constituent une des complications majeures de cette maladie. Nous rapportons le cas d'un patient qui s'est présenté en consultation pour douleur thoracique avec toux et crachats hémoptoïques. Le diagnostic d'une maladie de Behçet est retenu sur des arguments cliniques et confirmé par un angioscanner thoracique qui a objectivé des dilatations anévrysmales des bronches lobaires et segmentaires des deux artères pulmonaires. Devant l'urgence thérapeutique, un bolus de solumédrol sur trois jours est réalisé relayé par un bolus de cyclophosphamide. Après 12 mois de suivi, le patient rapporte une nette amélioration clinique. L'angio-scanner de contrôle note une disparition totale des anévrysmes des artères pulmonaires. L'atteinte thoracique de l'angio-Behçet est grave et met en jeu le pronostic vital, le traitement médical de ces anévrysmes à base d'immunosuppresseur permet une évolution favorable.

## Introduction

La maladie de Behçet est une Vascularite multisystémique d’étiologie obscure, caractérisée par des poussées d'inflammation aigue, séparées par des phases de rémission. L'angio-Behçet thoracique est en rapport avec le tropisme vasculaire bien connu de la maladie et les anévrysmes des artères pulmonaires constituent une des complications majeures de cette maladie. La maladie de Behçet touche surtout l'homme jeune associant une aphtose buccogénitale à des manifestations systémiques diverses. Le diagnostic de la maladie de Behçet repose surtout sur un faisceau d'arguments cliniques, tandis que le diagnostic de l'angio-Behçet repose essentiellement sur les examens tomodensitométriques. Nous présentons à travers cette observation, l'amélioration clinique et surtout la disparition totale des anévrysmes des artères pulmonaires chez un patient suivie pour une maladie de Behçet.

## Patient et observation

Un patient de 53 ans, sans antécédents pathologiques, se présentant en consultation pour douleur thoracique avec toux et crachats hémoptoïques. La symptomatologie remonte à un mois par l'installation d'une toux rebelle aux traitements symptomatiques qui s'est compliquée d'une hémoptysie de moyenne abondance. L'examen clinique trouve une pseudofolluculite dorsale, un aphte buccal, et des cicatrices d'aphtes génitaux. Le diagnostic d'une maladie de Behçet est évoqué et le patient a bénéficié d'un examen ophtalmologique détaillé qui est revenu normal. Après avoir éliminé l'origine tuberculeuse de l'hémoptysie par la réalisation de bronchoscopie avec biopsie et lavage bronchoalvéolaire, un angioscanner thoracique est réalisée, ce qui a objectivé des dilatations anévrysmales des bronches lobaires et segmentaire des deux artères pulmonaires.

Devant l'urgence thérapeutique, un bolus de solumédrol à raison de 15mg/kg/jour sur trois jours est réalisé relayé par un bolus de cyclophosphamide à raison de 700mg/m^2^. Après le traitement d'attaque le patient a bénéficié des cures de cyclophosphamide à la même dose à J15, J30, J60, J90et J120, avec une corticothérapie à raison de 1mg/kg/j en intercure, réduite progressivement après 4 semaines puis introduction de l'Azathioprine à raison de 2mg/kg/jour sans oublier la colchicine.

Le suivie est marqué par le tarissement des hémoptysies au bout de 1 mois, avec persistance de quelques pseudofolliculite au niveau dorsal. Après 12mois de traitement, le patient rapporte une nette amélioration clinique marquée par la non récidive des hémoptysies et la disparition des lésions pseudofolliculaire. L'angio-scanner de contrôle note une disparition totale des anévrysmes des artères pulmonaires.

## Discussion

C′est le dermatologue turque Hulusi Behçet qui décrivit pour la première fois en 1937 la classique triade uvéite, aphtose orale et aphtose génitale, évocatrice de la maladie qui porte depuis son nom [1]. La MB est caractérisée par une atteinte multisystémique d′étiologie inconnue, pour laquelle ont été incriminés des facteurs viraux, bactériens, génétiques, environnementaux et immunologiques [2].

La pathogénie de la maladie de Behçet est encore très discutée, elle fait intervenir plusieurs facteurs génétiques, infectieux et immunitaires [3]. Le substratum anatomique de cette maladie est une vascularite touchant aussi bien les veines que les artères [4], se traduisant par des thromboses et des anévrismes c'est des véritables aphtes vasculaires. Dans la maladie de Behçet, les vascularites expliquent une grande partie du processus pathologique. La participation vasculaire est actuellement considérée comme un signe critique de l’évolution clinique des patients présentant la maladie de Behçet. Selon les données disponibles, 7% à 29% des patients atteints de Behçet ont des lésions vasculaires, Ces atteintes vasculaires peuvent être des thromboses artérielles ou veineuses, des anévrismes ou pseudo-anévrismes, toutes ces anomalies pouvant s′associer. Au cours de la maladie de Behçet, les anévrysmes des artères pulmonaires sont considérés comme exceptionnels [5, 6]. Ces anévrysmes ont fait l'objet de quelques publications. Sur le plan clinique, ces anévrysmes se manifestent par des hémoptysies récidivantes de faible abondance comme ces le cas de notre patient. L'atteinte artérielle se produit chez 1% à 7% des patients présentant une maladie de Behçet mais peuvent être au premier rang du tableau clinique -comme le confirme notre cas- et causer des complications engageants le pronostic vital [7, 8]. L'artère le plus souvent affectée est l'aorte, suivie de l'artère pulmonaire [9].

**Figure 1:**
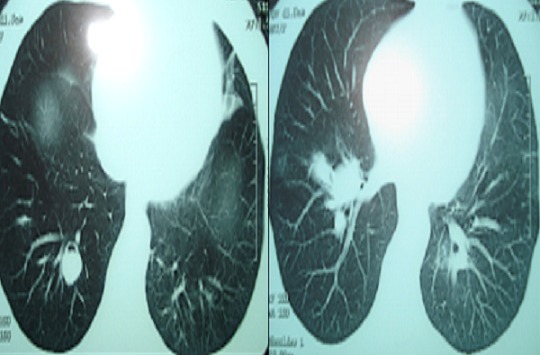
Coupes scannographiques d'un patient suivi pour Maladie de Behçet, montrant l'anévrisme des branches de l'artère pulmonaire.

**Figure 2:**
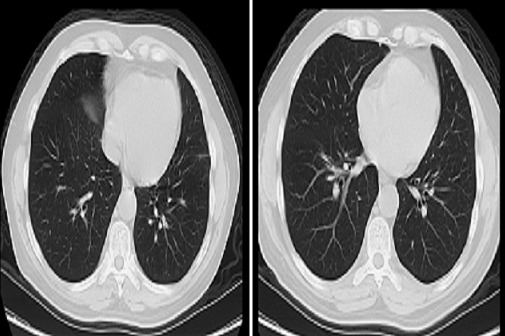
Coupes scannographiques chez le même patient suivi pour Maladie de Behçet, montrant la disparition des anévrismes des branches de l'artère pulmonaire après traitement médical.

Ces anévrysmes sont habituellement multiples, bilatéraux et de siège proximal intéressant les troncs et les bronches lobaires ou segmentaire des artères pulmonaires (Figure 1, Figure 2, Figure 3, Figure 4), et qui peuvent dans certaines circonstances se compliquer d'hémoptysies foudroyantes mettant en jeu le pronostic vital du patient.

Le traitement de la MB n′est pas bien codifié mais c′est en fonction de la sévérité des manifestations systémiques que peuvent être utilisés la colchicine, le thalidomide, les corticostéroïdes et les immunosuppresseurs [2].

En ce qui concerne l'atteinte vasculaire, le traitement chirurgical d'un anévrysme avec tous ses risques chez les patients présentant une maladie de Behçet a fréquemment comme conséquence une récidive anévrysmale [9]. Il consiste habituellement a une reconstruction utilisant des greffons vasculaires. Mais elle est souvent difficile et certains spécialistes préfèrent ne réaliser qu′une fermeture de la communication de l′anévrisme. Des néo-anévrismes ont été observés aux points de ponction vasculaire et en conséquence les explorations par artériographie devraient être évitées.

Ainsi, le traitement se base essentiellement sur la corticothérapie à forte dose et des bolus de Cyclophosphamide, tandis que l'usage des anticoagulants est formellement contre-indiqué vus la présence des anévrysmes qui peuvent se compliquer de ruptures, ce qui rejoint notre attitude thérapeutique dans notre cas.

Une revue complète de la littérature ne nous a permis de constater que le traitement des anévrysmes de l'artère pulmonaire au cours de la maladie de Behçet reste un sujet de controverse, vue les complications qui émaillent de ce traitement. De ce fait on propose cette observation qui met en évidence une alternative thérapeutique possible des anévrysmes au cours de la maladie de Behçet, bien sur en dehors de toute urgence vitale, et qui permet une rémission complète des anévrysmes pulmonaires au cours de la maladie de Behçet.

## Conclusion

L'atteinte thoracique de l'angio-Behçet est grave et met en jeu le pronostic vital, le traitement médical ces anévrysmes à base d'immunosuppresseur assure une évolution favorable. Notre observation permettra de lancer une étude à grande échelle pour codifier la prise en charge thérapeutique des anévrysmes de l'artère pulmonaire au cours de la maladie de Behçet.
